# Gluten-Free Alternative Grains: Nutritional Evaluation and Bioactive Compounds

**DOI:** 10.3390/foods8060208

**Published:** 2019-06-12

**Authors:** Serena Niro, Annacristina D’Agostino, Alessandra Fratianni, Luciano Cinquanta, Gianfranco Panfili

**Affiliations:** 1 Dipartimento di Agricoltura, Ambiente e Alimenti, Università degli Studi del Molise, Via De Sanctis, 86100 Campobasso, Italy; serena.niro@unimol.it (S.N.); a.dagostino@studenti.unimol.it (A.D.A.); panfili@unimol.it (G.P.); 2 Dipartimento Scienze Agrarie, Alimentari e Forestali, Università di Palermo, Viale delle Scienze 4, 90128 Palermo, Italy; luciano.cinquanta@unipa.it

**Keywords:** minor cereal, pseudocereal, bioactive compound, gluten-free grain, tocols, carotenoids

## Abstract

Interest in gluten-free grains is increasing, together with major incidences of celiac disease in the last years. Since to date, knowledge of the nutritional and bioactive compounds profile of alternative gluten-free grains is limited, we evaluated the content of water-soluble (thiamine and riboflavin) and liposoluble vitamins, such as carotenoids and tocols (tocopherols and tocotrienols), of gluten-free minor cereals and also of pseudocereals. The analysed samples showed a high content of bioactive compounds; in particular, amaranth, cañihua and quinoa are good sources of vitamin E, while millet, sorghum and teff (*Eragrostis tef*, or William’s Lovegrass) are good sources of thiamine. Moreover, millet provides a fair amount of carotenoids, and in particular of lutein. These data can provide more information on bioactive compounds in gluten-free grains. The use of these grains can improve the nutritional quality of gluten-free cereal-based products, and could avoid the monotony of the celiac diet.

## 1. Introduction

Celiac disease is a chronic systemic, autoimmune disorder in genetically-predisposed individuals, triggered by exposure to dietary gluten, and resulting in mucosal inflammation, villous atrophy and crypt hyperplasia [1]. It is characterised by an abnormal immune reaction consisting of an excessive response of the immune system to a group of cereal proteins, called prolamines (gliadin, hordein, sekalina, avenin), which are found in wheat, barley, rye and oats. Celiac disease affects approximately 1% of the world population, and it has significantly increased due to an underestimation, since it is often left undiagnosed [2]. The only treatment for people with the celiac problem is the adherence to gluten-free foods for their whole lifetime.

Several studies demonstrated that sticking to a gluten-free diet for a lifetime can lead to a nutritional imbalance in celiac subjects, such as a malabsorption of nutrients, and deficiencies of several vitamins and minerals. These deficiencies are due both to the phenomena of malabsorption at the intestinal level, and to the monotony of a diet based mainly on rice and maize [3,4,5,6].

Recently, more attention has been given to gluten-free minor cereals and pseudocereals as alternatives to those conventionally used for celiacs. Many of them have been defined as “orphan crops” or “underutilised crops”; they are indigenous crops scarcely documented and rarely used by food industries [7]. Many underutilised crops are relatively more drought-tolerant than most major cereals; they play a significant role in many developing countries, providing food security and income to resource-poor farmers [8]. 

Gluten-free alternative sources studied in this work include minor cereals (sorghum, teff, millet and wild rice), and pseudocereals (quinoa, cañihua, chia, and amaranth). These grains are mainly consumed as flours and seeds, which can be added to preparations such as soups, yogurt, cakes, breads and others cereal-based products; nevertheless, any commercialisation of these products is still quite limited in the Italian market. Some of these are a source of nutrients and bioactive compounds that could improve the nutritional quality of gluten-free products. 

Carotenoids are a significant group of bioactive compounds with health promoting properties [9,10] and are responsible for the colour of a wide variety of grains [11]. Some carotenoids are the precursors of retinol (vitamin A), and are very strong natural antioxidants. Carotenoids are known to be efficient physical and chemical quenchers of singlet oxygen, as well as potent scavengers of other reactive oxygen species [9]. Vitamin E is a natural antioxidant comprising two groups of vitamers, tocopherols and tocotrienols, occurring in eight forms: α-tocopherol (α-T), β-tocopherol (β-T), γ-tocopherol (γ-T), and δ-tocopherol (δ-T) and α-tocotrienol (α-T3), β-tocotrienol (β-T3), γ-tocotrienol (γ-T3), and δ-tocotrienol (δ-T3). Vegetable oils are the main tocol sources, however, substantial amounts of these compounds are also reported in most cereal grains [12,13,14]. The potential health benefits of tocols include the prevention of certain types of cancer, heart diseases and other chronic diseases [15,16]. Thiamine (B1) is one of the major water-soluble vitamins, as it plays an important role as a co-factor of several key enzymes involved in the carbohydrate metabolism and defence mechanism [17]. It can be found in moderate amounts in all foods: Nuts and seeds, legumes, wholegrain/enriched cereals and breads, as well as pork [18]. Thiamine deficiency is rare in healthy individuals in food-secure settings, where access to thiamine-rich foods ensures adequate intakes [19]. Riboflavin (B2) is a precursor of the co-enzymes flavin mononucleotide (FMN; riboflavin phosphate) and flavin adenine dinucleotide (FAD), which are components of oxidases and dehydrogenases. It is also important for skin health and normal vision, and can be found in whole cereals, breads, leafy green vegetables and milk products [18].

To date, the evaluation of nutritional and bioactive compound profiles of alternative gluten-free grains is limited, if not lacking [20,21,22,23]. These researches are of a great importance in order to formulate gluten-free cereal-based products with a higher nutritional value. Thus, in this work, samples of minor cereals and pseudocereals commercialised in Italy have been characterised for their nutritional value, with a particular focus on some bioactive compounds, such as carotenoids, tocols, thiamine and riboflavin, in order to increase the awareness of their nutritional profile. Moreover, data coming from this study may be included in food nutrient databases.

## 2. Material and Methods

### 2.1. Sample Collection and Preparation

Thirty one different minor cereals and pseudocereals were bought in Italian specialised shops (Table 1). Different brands were considered for each grain. Grains were grounded with a refrigerated IKA A10 laboratory mill (Staufen, Germany), then carefully mixed and stored at −20 °C until analysis. Each sample was analysed in triplicate.

### 2.2. Chemical Analysis

#### 2.2.1. Proximate Analysis

Moisture, ash, fat, and protein contents were determined using an ICC standard procedure [24]. Briefly, moisture was determined using an oven set at 130 °C, and ash was quantified using a muffle furnace set at 525 °C. The protein content was determined though the Kjeldhal method (N × 6.25), and lipids were determined by the Soxhlet method. Carbohydrates plus fibre were calculated as a difference, using the following equation: (100 − (% moisture + % lipids + % proteins + % ash)).

#### 2.2.2. Carotenoid Analysis

Carotenoid extraction was carried out using the saponification method reported by Panfili et al. [14]. About 0.2 g of milled sample was weighed and placed in a screw-capped tube. Then, 5 mL of ethanolic pyrogallol (60 g/L) was added as an antioxidant, followed by 2 ml of absolute ethanol, 2 mL of sodium chloride (10 g/L) and 2 mL of potassium hydroxide (600 g/L). The tubes were placed in a 70 °C water bath and mixed every 5–10 min during saponification. After alkaline digestion at 70 °C for 45 min, the tubes were cooled in an ice bath, and 15 mL of sodium chloride (10 g/L) were added. The suspension was then extracted twice with 15 mL portions of n-hexane/ethyl acetate (9:1, *v*/*v*). The organic layers, containing carotenoids, were collected and evaporated to dryness; the dry residue was dissolved in 2 mL of isopropyl alcohol (10%) in *n*-hexane. A HPLC Dionex (Sunnyvale, CA) analytical system, consisting of a U6000 pump system and a 50 μL injector loop (Rheodyne, Cotati) was used. The chromatographic separation of the compounds was achieved by means of a 250 mm × 4.6 mm i.d., 5 µm particle size, Kromasil Phenomenex Si column (Torrance, CA, USA). The mobile phase was *n*-hexane/isopropyl alcohol (5%) at a flow rate of 1.5 mL/min. Spectrophotometric detection was achieved by means of a diode array detector set in the range of 350–500 nm. Peaks were detected at 450 nm. Carotenoids were identified through their spectral characteristic, and comparison of their retention times with known standard solutions. Data were stored and processed by a Dionex Chromeleon Version 6.6 chromatography system (Sunnyvale, CA, USA). All-trans-β-carotene and lutein were obtained from Sigma Chemicals (St. Louis, MO, USA); zeaxanthin and β-cryptoxanthin were obtained from Extrasynthese (Z.I. Lyon-Nord, Genay, France). 

#### 2.2.3. Tocol Analysis

Tocols were determined after the same saponification method described for carotenoids. An aliquot of the carotenoid extract was collected and evaporated to dryness, and the dry residue was dissolved in 2 mL of isopropyl alcohol (1%) in *n*-hexane, and was analysed by HPLC, under normal phase conditions, using a 250 × 4.6 mm i.d., 5 mm particle size Kromasil Phenomenex Si column (Torrance, CA, USA) [14]. Fluorometric detection of all compounds was performed at an excitation wavelength of 290 nm and an emission wavelength of 330 nm by means of an RF 2000 spectrofluorimeter (Dionex, Sunnyvale, CA, USA). The mobile phase was *n*-hexane/ethylacetate/acetic acid (97.3:1.8:0.9 *v*/*v*/*v*), at a flow rate of 1.6 mL/min [14,25]. Compounds were identified by a comparison of their retention times with those of known available standard solutions, and quantified through the calibration curves of the standard solutions. The concentration range was 5–25 μg/mL for every tocol standard. Vitamin E activity was expressed as Tocopherol Equivalent (T.E.) (mg/100 g of fresh weight f.w.), calculated as reported by Sheppard et al. [26].

#### 2.2.4. Thiamine and Riboflavin Analysis

Thiamine and riboflavin were extracted as in Hasselman et al. [27]. Briefly, samples were placed in 100 mL volumetric flasks containing 20 mL of 0.1 N HCl and heated in a water bath at 100 °C for 30 min. After cooling at room temperature, the pH of the samples was adjusted to 4.5 with 2.5 M NaOAc. Following the addition of 0.2 mL of aqueous Clara-Diastase (50 mg/mL), these samples were incubated for 3 h at 37 °C. After cooling, the samples were brought up to 25 mL with distilled water. Then these same samples were centrifuged and filtered through a 0.45 µm filter. Thiamine was converted to thiochrome by adding 1.25 mL of 1% potassium ferricyanide in 15% aqueous NaOH to 2.5 mL of filtered extract. After 1 min for oxidation, 0.25 mL of 85% H_3_PO4 was added. The extract was purified on a Sep-Pak C18 cartridge. The cartridge was washed with 5 mL MeOH, followed by 5 ml of 0.05 M NH_4_OAc (adjusted to pH 5.0 (acidic) with HOAc). The sample (5 mL) was loaded into a Sep-Pak C18 cartridge, and then the cartridge was washed with 0.05 M NH_4_OAc and, finally, the vitamins were eluted with 5 mL mobile phase. Extracts were separated by a HPLC Dionex (Sunnyvale, CA, USA), with a U3000 pump and an injector loop (Rheodyne, Cotati). Separation was made at a flow rate of 0.8 mL/min with Methanol: NaOAc (40:60 *v*/*v*) as a mobile phase, by using a 5 µm C18 Luna, Phenomenex (Torrance, CA, USA) stainless steel column (250 × 4.6 mm i.d.). Fluorometric detection was performed at an excitation wavelength of 366 nm and an emission wavelength of 453 nm for thiamine, and an excitation wavelength of 453 nm and an emission wavelength of 580 nm for riboflavin, by means of an RF 2000 spectrofluorimeter (Dionex, Sunnyvale, CA, USA). Data were processed by a Dionex Chromeleon Version 6.6 chromatography system (Sunnyvale, CA, USA). Thiamine and riboflavin were compared with known available standards, and identified considering their retention times and relative elution order. Thiamine and riboflavin standards were obtained from Sigma Chemicals (St. Louis, MO, USA).

## 3. Results and Discussion

### 3.1. Nutritional Composition

The nutritional composition of analysed minor cereals and pseudocereals is shown below in Table 2. 

The composition of the chia seeds notably differs from all the other cereal and pseudocereal samples, showing high concentrations of fats (35.4 g/100 g), proteins (21.5 g/100 g) and ash (4.5 g/100 g). These values are similar to those observed by other authors for the chia seeds [28]. In general, wild rice and pseudocereals are a good source of protein. Taking European law into account [29], wild rice, all quinoa seeds, cañihua and amaranth can be declared in a label with the claim “source of protein”, since they contain at least 12 g of protein per 100 g. Chia seeds can be declared with a “high protein content”, since they contain at least 20 g of protein per 100 g. The fat content was significantly higher for pseudocereals, if compared to minor cereals. Wild rice shows the lower fat content (1.2 g/100 g).

### 3.2. Carotenoids

Table 3 shows the carotenoid amounts of analysed samples. Carotenoids content (μg/100 g dry weight d.w.) varied significantly from 22 μg/100 g in amaranth to 763 μg/100 g in millet. In all gluten-free grains the main compounds are lutein and zeaxanthin. A comparison with the literature related to the HPLC analysis of carotenoids is very difficult, since the available few data are obtained by different methods, and these pigments may vary depending on genotype and location. The total carotenoid content of millet, wild rice, quinoas and cañihua is comparable with that of wheat (about 305 μg/100 g for durum and about 150 μg/100 g for soft wheat) [12,30], and of pigmented rice (460–50 μg/100 g) [31], but it is significantly lower than that of maize (about 1110 μg/100 g) [30,32]. Among minor cereals, literature data are reported only for sorghum [33], where the authors found an average amount of 20 µg/100 g as the sum of lutein and zeaxanthin, with a high variability among the different genotypes.

In the present study, the variability of the total carotenoid content within the same cereal (expressed by the coefficient of variability, CV%), is from 4% in millet to 26% in pigmented quinoa. This variability may be due to genetic, pedoclimatic and varietal factors [34]. Regarding pseudocereals, results for chia are similar to those obtained in the work of da Silva et al. [28]. Significant differences between white and pigmented quinoas were found for total carotenoids, due to the different lutein amounts, as also observed by Tang et al. [35], who indicate a direct correlation between the higher total carotenoid content and the darkness of the seed coat.

### 3.3. Tocols

The characterisation of tocols in minor cereals and pseudocereals is reported in Table 4. Except for wild rice, which shows a minor content of total tocols (TC) (about 0.4 mg/100 g), the TC of minor cereals and amaranth are comparable with that of wheat, maize and rice (about 3.5–7.0, 6.0–7.0 and 2.3–2.7 mg/100g, respectively) [12,14,36] while, for the remaining pseudocereals, these values are significantly higher. Among minor cereals, teff shows the highest amount of total tocols (6 mg/100g d.w.), followed by millet and sorghum with about 4 and 3 mg/100g respectively.

Except for wild rice, where α-tocopherol is the prevalent isomer, the main tocopherol isomer is γ-tocopherol, which represents the 92%, 72% and 75% of the total content in teff, millet and sorghum, respectively. For pseudocereals, the highest content of total tocols was found in cañihua (about 18 mg/100 g), followed by chia seeds (about 14 mg/100 g d.w.) and quinoas, with an average of 9.1 mg/100 g d.w. Contrarily to carotenoids, among all analysed quinoa seeds, all of the found vitamers did not show significant qualitative and quantitative differences. Amaranth is the pseudocereal with the lowest total tocol amounts (about 6 mg/100g). For chia, cañihua and quinoa the predominant isomer is γ-tocopherol (94%, 69% and 64% of total tocols), while for amaranth the prevalent isomer is β-tocopherol, which represents 54% of the total tocols.

γ-Tocopherol has also been found as the main vitamer in quinoa and chia in other works [28,35,37]. References for tocols are not available for all analysed gluten-free grains and, where present, they show similar results in millet and sorghum [3,23]. Moreover a comparison with the literature data related to tocol analysis is very difficult, for the same reasons already explained for carotenoids.

Table 4 also reports values of vitamin E activity provided by 100 g of product, expressed as Tocopherol Equivalent (T.E.) (mg/100 g product) [26]. Taking into account the Recommended Daily Allowance (RDA) for vitamin E, which is of 12 mg/day [38], 100 g of amaranth contribute to 22% of the RDA, while quinoas and cañihua approximately to 35% of the RDA, so as to be declared in a label as a “source of vitamin E”. A portion of these pseudocereals (70 g) contributes approximately to 15% of the RDA for amaranth and to 25% of the RDA for quinoas and cañihua.

### 3.4. Thiamine and Riboflavin 

Table 5 reports the values of the thiamine and riboflavin of analysed grains. The concentrations of thiamine are different between minor cereals and pseudocereals, except for wild rice. In whole wheat grains about 0.40 mg/100g are found in the literature [39,40]. Low values of riboflavin were found for all samples, except for wild rice, with values comparable to those of whole wheat grains and maize (0.15 and 0.20 mg/100g, respectively) [39,40].

Taking into account the Recommended Daily Allowance (RDA) for thiamine, which is of 1.1 mg/day [38], 100 g of teff would contribute to approximately 17% of the RDA, while 100 g of millet and sorghum to 23% of the RDA, so as to be declared in a label as a “source of thiamine”. A portion of 80 g contributes approximately to 16% of the RDA for teff and to 20% of the RDA for millet and sorghum.

## 4. Conclusion

Naturally gluten-free products are corn, rice, potatoes, soybean, millet, buckwheat, tapioca, amaranth, cassava, lentils, beans, sago, sorghum, nuts, as well as meat, fruit and vegetables. Among these, cereals and pseudocereals are becoming increasingly important. This work confirms that minor cereals and pseudocereals are an important source of bioactive compounds. In particular, wild rice and all analysed pseudocereals are good sources of protein. Taking into account the Recommended Daily Allowance (RDA) for vitamins established by the Commission of the European Communities, amaranth, cañihua and quinoa can be declared on the label as a source of vitamin E, the main antioxidant found in cells involved in the prevention of several diseases. Moreover, millet, sorghum and teff can be declared on the label as a potential source of thiamine. Millet also provides a fair amount of lutein. In the light of these results, it is possible to use the combined mix of these flours in order to improve the nutritional value of cereal-based gluten-free products.

## Figures and Tables

**Table 1:** List of analysed gluten-free grains.

Minor Cereals	Samples (*n*)
Millet (*Panicum miliaceum* L.)	6
White Sorghum (*Sorghum bicolor* L.)	3
Teff (*Eragrostis tef* (Zucc.) Trotter )	4
Wild rice (*Zizania aquatica* L.)	2
**Pseudocereals**	
White quinoa *(Chenopodium quinoa* Willd.)	3
Pigmented quinoas (red and black) (*Chenopodium quinoa* Willd.)	4
Cañihua *(Chenopodium pallidicaule)*	3
Amaranth *(Amaranthus* spp.)	3
Chia *(Salvia hispanica* L.)	3

**Table 2:** Nutritional composition of gluten-free grains (g/100 g).

	Minor Cereals				Pseudocereals			
	Millet	Sorghum	Teff	Wild Rice	Quinoa (White and Pigmented)	Cañihua	Amaranth	Chia
Moisture	12.7 (2.0) ^a^	12.5 (6.9)	11.5 (1.4)	10.5 (0.4)	11.5 (9.8)	8.6 (5.7)	11.0 (1.0)	8.4 (6.7)
Ash	1.0 (63.0)	1.4 (8.6)	2.3 (5.6)	1.8 (7.2)	2.2 (3.0)	2.4 (6.6)	2.3 (8.7)	4.5 (2.7)
Protein	11.7 (3.3)	9.0 (0.1)	11.7 (1.7)	12.4 (6.1)	12.9 (1.5)	14.1 (2.6)	13.8 (3.4)	21.5 (7.6)
Fat	4.4 (0.4)	2.6 (26.9)	2.4 (4.1)	1.2 (4.5)	5.8 (12.0)	8.4 (1.1)	6.1 (5.7)	35.4 (2.1)
Carbohydrate + Fibre *	70.2	74.5	72.1	74.1	67.6	66.8	66.8	30.2

* Calculated by difference; ^a^: coefficient of variability.

**Table 3:** Carotenoid composition in gluten-free grains (μg/100 g d.w.).

Carotenoids	Minor Cereals				Pseudocereals				
	Millet	Sorghum	Teff	Wild Rice	White Quinoa	Pigmented Quinoas	Cañihua	Amaranth	Chia
β-Carotene	19.8 (15.0) ^a^	9.86 (10.0)	7.8 (20.0)	6.23 (10.0)	12.3 (10.0)	23.6 (23.0)	20.2 (28.0)	tr	12.4 (10.0)
β-Criptoxanthin	20.0 (30.0)	nd	tr	tr	tr	tr	tr	nd	nd
Lutein	535.5 (3.4)	11.2 (64.0)	36.45 (30.0)	196.2 (36.6)	85.6 (1.3)	265.2 (33.0)	325.3 (0.1)	19.8 (5.0)	tr
Zeaxanthin	188.3 (10.0)	28.9 (10.0)	18.4 (40.0)	9.7 (10.0)	11.2 (11.0)	13.2 (30.0)	40.2 (4.2)	2.2 (11.3)	33.5 (10.0)
Total Carotenoid	763.1 (4.0)	50.46 (8.0)	62.6 (28.0)	212.3 (8.0)	109.1 (11.0)	302.0 (26.0)	385.7 (10.0)	22.0 (10.0)	45.9 (8.0)

^a^: Coefficient of variability; nd: not detectable; tr: traces.

**Table 4:** Tocol composition in gluten-free grains (mg/100g d.w.)

Tocols	Minor Cereals				Pseudocereals			
	Millet	Sorghum	Teff	Wild Rice	Quinoa (White and Pigmented)	Cañihua	Amaranth	Chia
α-T	0.16 (6.2) ^a^	0.60 (83.0)	0.11 (18.2)	0.13 (11.5)	2.86 (9.58)	4.2 (35.7)	1.28 (44.5)	0.33 (33.3)
β-T	0.06 (16.6)	0.08 (62.5)	0.06 (20.0)	0.10 (13.0)	0.11 (23.0)	0.28 (21.0)	3.43 (46.0)	nd ^b^
γ-T	2.73 (47.2)	2.32 (41.4)	5.52 (8.3)	0.10 (1.0)	5.9 (8.3)	12.50 (4.3)	0.30 (36.7)	13.59 (21.5)
δ-T	0.45 (29.0)	0.03 (33.0)	0.14 (14.0)	nd	0.22 (1.0)	0.40 (5.0)	1.28 (35.0)	0.38 (34.0)
α-T3	nd	nd	nd	nd	nd	nd	nd	nd
β-T3	0.12 (50.0)	nd	nd	0.03 (16.6)	tr	nd	nd	nd
γ-T3	0.04 (25.0)	nd	0.15 (73.0)	nd	tr	nd	nd	0.13 (23.0)
δ-T3	0.24 (45.8)	nd	nd	nd	nd	nd	nd	nd
Total tocols	3.80 (45.0)	3.09 (51.0)	5.99 (5.0)	0.36 (1.0)	9.10 (8.0)	18.06 (3.9)	6.31 (42.0)	14.43 (22.0)
T.E.	0.43 (28.0)	0.78 (82.0)	0.56 (21.0)	0.17 (5.9)	3.62 (1.0)	4.5 (2.0)	2.7 (33.0)	1.6 (24.0)

^a^: Coefficient of variability; ^b^: Not detectable; tr: traces; T.E.: Tocopherol equivalent (mg/100g f.w.).

**Table 5:** Thiamine and riboflavin content in gluten-free grains (mg/100g d.w.).

	Thiamine (mg/100g d.w.)	CV%	%RDA (1.1 mg/100g f.w.)	Riboflavin (mg/100g d.w.)	CV%	%RDA (1.4 mg/100g f.w.)
**Minor Cereals**						
Millet	0.28	49	23	0.02	25	1
Sorghum	0.28	61	23	tr	75	-
Teff	0.22	35	17	0.02	15	1
Wild rice	0.08	28	6	0.17	3	11
**Pseudocereals**						
Quinoa (white and pigmented)	0.13	50	9	0.02	32	1
Cañihua	0.04	6	3	0.09	8	6
Amaranth	0.03	23	3	0.01	20	1
Chia	0.06	2	5	0.02	20	1

tr: traces.
